# A likelihood ratio framework for inferring close kinship from dynamically selected SNPs

**DOI:** 10.3389/fgene.2025.1635734

**Published:** 2025-07-23

**Authors:** Jianye Ge, Bruce Budowle, Michael Cariaso, Kristen Mittelman, David Mittelman

**Affiliations:** ^1^ Othram Inc., The Woodlands, TX, United States; ^2^ Department of Forensic Medicine, University of Helsinki, Helsinki, Finland; ^3^ Forensic Science Institute, Radford University, Radford, VA, United States

**Keywords:** forensic genetic genealogy, single nucleotide polymorphism, likelihood ratio, kinship analysis, identity by descent, relationship testing, whole genome sequencing

## Abstract

Forensic genetic genealogy (FGG) is a force-multiplier for human identification, leveraging dense single nucleotide polymorphism (SNP) data to infer relationships through identity by descent (IBD) segment analysis. Although powerful for investigative lead generation, broad adoption of SNP-based identification methods by the forensic community, especially medical examiners and crime laboratories, necessitates likelihood ratio (LR)-based relationship testing, to align with traditional kinship testing standards. To address this gap, a novel method was developed that incorporates LR calculations into FGG and SNP testing workflows. This approach is unique in that it dynamically selects unlinked, highly informative SNPs based on configurable thresholds for minor allele frequency (MAF) and minimum genetic distance for a robust and reliable analysis. Employing a curated panel of 222,366 SNPs from gnomAD v4 and data from the 1,000 genomes project, high accuracy in resolving relationships up to second-degree relatives can be achieved. For example, a subset of 126 SNPs (MAF > 0.4, minimum genetic distance of 30 cM) yielded 96.8% accuracy and a weighted F1 score of 0.975 across 2,244 tested pairs. This LR-based methodology enables forensic laboratories to select informative SNPs and integrate modern genomic data with existing accredited relationship testing frameworks, providing critical statistical support for close-relationship comparisons and enhances the rigor of FGG- and SNP-based human identification applications.

## Introduction

Advances in genomic analyses, particularly massively parallel sequencing (MPS), have enabled unprecedented high throughput SNP analyses which have fostered the development of Forensic Genetic Genealogy (FGG). This subdiscipline of forensic genetics exploits genome-wide dense single nucleotide polymorphism (SNP) data generated by whole genome sequencing (WGS) or microarrays to determine near and distant kinship relationships. While targeted sequencing, in theory, can be used in forensic contexts to interrogate selected SNPs at lower cost (although current commercial forensic options are relatively costly), whole genome sequencing (WGS) offers a more comprehensive approach, capturing significantly more genetic variation and enabling deeper kinship inference. As sequencing costs continue to decline, WGS is becoming increasingly preferred in FGG for its broader utility and richer dataset (Mandape, et al., 2024). The kinship relationships can facilitate investigative leads for identification of human remains and source attribution of donors of crime scene DNA evidence more effectively than current forensic genetic methods (Dowdeswell, 2023; Kling, 2019; Kling and Tillmar, 2019; Mandape et al., 2024; Budowle et al., 2024). Kinship associations typically are determined using either IBS (identity by State) or IBD (identity by Descent) segmented-based methods (Henn et al., 2012; Browning and Browning, 2011; Browning and Browning, 2013; Huff et al., 2011; Lipatov et al., 2015; Staples et al., 2016; Kaplanis et al., 2018; Ramstetter et al., 2018; Morimoto et al., 2018; Seidman et al., 2020). While these approaches provide high accuracy, they differ from the likelihood ratio (LR)-based framework traditionally used in kinship analysis, which underpins the validity and acceptance of short tandem repeat (STR) interpretation methods in forensic applications. LR-based statistics are routinely employed to support identifications, making them a critical component of forensic practice. To enable the forensic community to leverage SNP data, an analogous LR-based framework is needed that offers the flexibility, rigor, interpretability, and validity required for broad acceptance. Accordingly, an LR-based interpretation method tailored to whole genome sequencing (WGS) data for SNP selection and relationship inference was developed, with a focus on pairwise comparisons up to second degree relatives.

Like traditional kinship analysis, the approach described herein calculates the likelihoods of each selected SNP for specific relationships, with LRs obtained by comparing the likelihoods of genetic data under alternative relationships. Assuming independence among SNPs, the cumulative LR is calculated by multiplying the LR values for each individual SNP. Therefore, selecting the most informative SNPs is critical to the success of the method. Our approach is unique in that it enables dynamic SNP selection in tandem with likelihood ratio (LR) calculations compared with traditional kinship software that relies on fixed, pre-selected markers. This dynamic integration allows for greater flexibility and improved performance when working with WGS data. A straightforward approach was used to refine a subset of SNPs from the hundreds of thousands to millions identified by WGS, prioritizing high minor allele frequency (MAF) markers observed in each case. These markers offer high discrimination power for relationship inference. Subsequently, the data are further subset to include SNPs located in genomic regions identified by Genome-in-a-Bottle as being easy to sequence or genotype (Dwarshuis et al., 2024). This subset is then further refined by selecting only SNPs that exhibit nominal or no linkage and linkage disequilibrium (LD). This approach provides a curated panel of SNPs with high MAF for robust LR support for true close kinship relationships, particularly up to the second degree.

Indeed, Yousefi et al. (2018) demonstrated in a Dutch sample population that 50 independent SNPs (MAF > 0.2; average MAF = 0.35) are sufficient to yield low probabilities of identity (or population match probabilities) of 6.9 × 10^−20^ and 1.2 × 10^−10^ for unrelated individuals and siblings, respectively. Chakraborty et al. (1999) estimated that approximately 40 SNPs (MAF ∼ 0.5) could achieve random match probabilities around 10^−15^ and 33 SNPs (MAF ∼ 0.5) would be required to reach an exclusion probability of 99.9% in a typical trio paternity case. Furthermore, the higher the MAF for a suite of binary SNPs (i.e., approaching the highest heterozygosity of 0.5) is globally, the lower are the effects of population substructure. Moreover, with high heterozygosity any positive predictive power with binary SNPs for associations with private genetic information would be negligible (Budowle and van Daal, 2008).

Here, the validation of a statistical approach, KinSNP-LR (version 1.1), is described for computing LRs based on WGS generated SNP data. Instead of *a priori* selecting a fixed panel of informative SNPs, the first SNP on an end of a chromosome passing the MAF threshold is selected from a large candidate panel and the next SNP at a specified genetic distance (e.g., 30–50 centimorgans (cM)) and meeting the MAF criterion is selected and so on across the genome. The LRs for multiple relationships are calculated based on the methods described in Thompson (1975), Ge et al. (2010), and Ge et al. (2011). This approach maximizes the number of SNPs with little to no linkage and LD selected in a case-specific manner.

## Materials and methods/implementation

### Empirical genomic data

A large, preselected SNP panel (222,366 SNPs) from gnomAD v4 (Chen et al., 2024) was used as the data foundation for this validation study (details on this panel can be found in Supplementary Material A). The SNP allele frequencies and genetic distances between the SNPs in this panel will be used in SNP selection and likelihood calculation in the kinship analysis. The SNPs in the panel were filtered and obtained by quality control, MAF, and “Not in all difficult regions” regions. The data contain nine populations: Admixed America, African, Ashkenazi Jewish, East Asian, Finnish, Non-Finnish European, Middle Eastern, South Asian, and Remaining Individuals (includes Amish). Five major populations: African (AFR), Admixed American (AMR), East Asian (EAS), South Asian (SAS), and Non-Finnish European (NFE), were selected in validating KinSNP-LR. More details about preparing this SNP panel can be found in the Supplementary Material.

In addition, the 1,000 genomes project data contain 3,202 whole genome sequenced samples with many closely related pairs, and these related pairs were used to validate the methodology. Each sample in the 1,000 genomes data was converted using the GRCh38 coordinate positions into a tab-separated text format, but only for the SNPs contained in the preselected gnomAD panel. After removing uncertain relationships, there are 1,200 parent-child, 12 full-sibling, and 32 second degree pairs in the 1,000 genome project. Unrelated pairs were randomly selected in the populations. The validation study described herein used data in GRCh38 coordinates.

### Simulation data

Pedigrees and phased genotypes simulations were performed using Ped-sim (v1.4) (Caballero et al., 2019) with the unrelated individuals in the ASW (Americans of African Ancestry in SW United States), CEU Utah Residents with Northern and Western European Ancestry), CHB (Han Chinese in Beijing, China), and MXL (Mexican Ancestry from Los Angeles, United States) populations in the 1,000 genomes project. Each population represents one of four major continental groups (African, East Asian, European, and Admixed American); thus a broad genetic diversity is captured as opposed to simulating all 26 subpopulations, many of which are closely related groups.

Only the SNPs in the preselected SNP panel from gnomAD v4 were used in the simulations, and only unrelated individuals in each of the populations were used as founders in the simulations. The unrelated relationships were confirmed with IBIS (Seidman et al., 2020) with a maximum total shared IBD segment of 40 cM.

**FIGURE 1:**
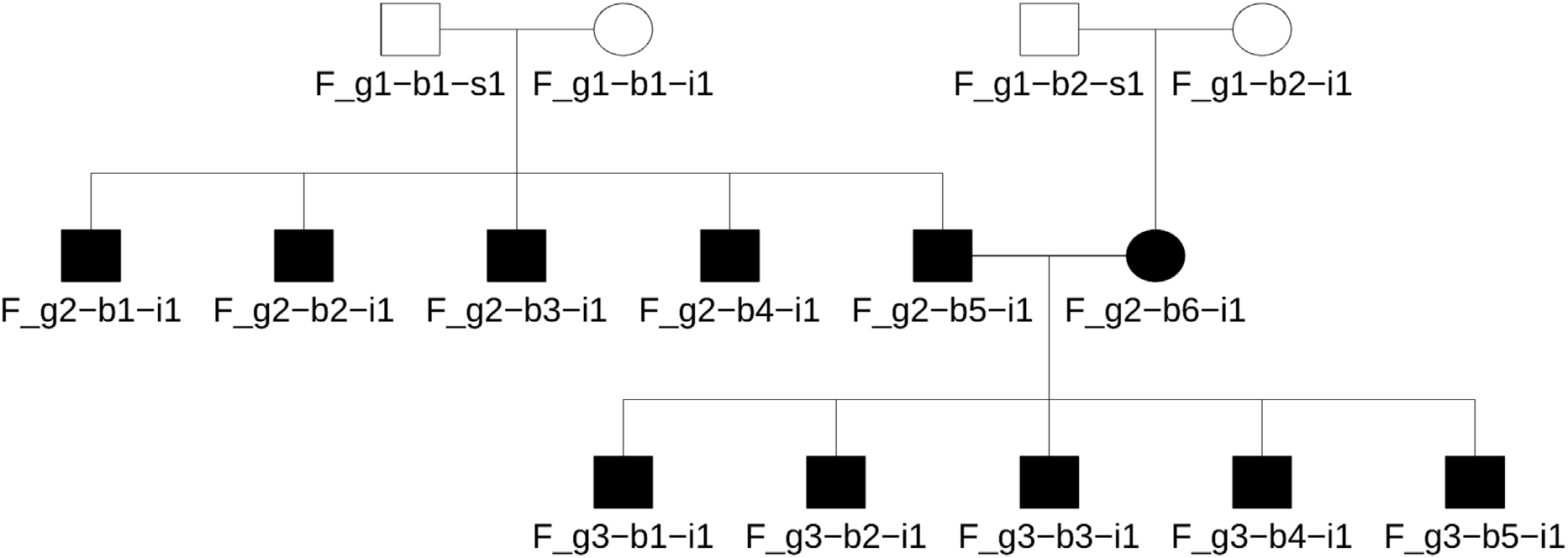
The simulated family tree using unrelated founders in the 1,000 genomes data. This pedigree includes first to second degree relationships and four unrelated founders. The same format was used for all populations studied herein.

Briefly, Ped-sim simulated 50 families as shown in Figure 1, in which there were three generations with second degree being the most distant relationship. Each family has 22 parent-child pairs, 20 sibling pairs, 40 second degree pairs, and 22 unrelated pairs. In the simulation, descendants inherit recombinant chromosomes, one from each parent, according to the specified pedigree. Recombination follows an interference model and applies sex-average genetic maps. Chromosome segments are tracked through the inheritance process, and genetic markers from the founders are overlaid on the corresponding segments to produce whole genome data. Simulations were conducted with true IBD segments and founder sample identifiers recorded with zero missing genotype call rate, zero opposite homozygote errors, and various genotyping errors (i.e., 0.001, 0.01, and 0.05). In the simulation, the keep_phase flag was enabled, using high-resolution sex average genetic maps from the Broad Institute (see Data availability for details) and chromosome interference maps (nu_p_campbell_X.tsv) from Campbell et al. (2015).

For LR calculations, the allele frequencies from the corresponding gnomAD major population were used. For example, gnomAD Non-Finnish European frequencies were used for the CEU pairwise LR calculations.

### Candidate SNP selection

To maximize information content, the SNPs with MAF higher than a threshold (e.g., 0.4) in each individual population, were selected for relationship tests. Further, only unlinked SNPs were selected with a Minimum Genetic Distance (MGD) cM threshold, such as 30 cM. This selection is necessary for following traditional LR-based kinship analyses that require independent markers to calculate cumulative likelihoods by directly multiplying the likelihood of each marker. LD tends to decay over distances of approximately 1 cM or less (Ardlie et al., 2002), and thus, if the MGD is greater than 1 cM, LD between SNPs may be ignored.

Genetic distances between SNP pairs were calculated based on the sex averaged genetic map. Genetic distance maps of both GRCh38/HG38 and GRCh37/HG19 are available at Broad Institute (see Data availability for details). Since the input profile only contains the SNP names and physical positions in the chromosomes, the SNPs’ physical positions were mapped to their genetic positions, so genetic distance in cM can be obtained between two SNPs on the same chromosome. The positions of the SNPs that are not in the genetic map were interpolated linearly.

To maximize the number of selected markers, the first SNP on an end of a chromosome passing the MAF threshold is selected from a large candidate panel, and the next SNP at a specified genetic distance (i.e., MGD) and meeting the MAF criterion is selected and so on across the genome. This dynamic SNP-selection algorithm applies a high MAF filter to a large candidate panel, then sweeps each chromosome once from an end inward, greedily choosing the first marker and every subsequent marker that lies at least a certain centimorgans (defined by the selected MGD) distance from the last one selected. Because all retained SNPs have similarly high MAF, information content per marker is nearly uniform, so simply maximizing the count within the distance constraint maximizes cumulative power. The procedure runs in linear time after sorting, yet it approximates the optimal distance-constrained subset-selection problem, which is NP-hard for exact solutions, and sufficient for forensic and association applications. The pseudocode of this dynamic SNP selection algorithm can be found in the Supplementary Material B.

### Kinship analysis

Once the SNPs are selected, the pairwise likelihood(s) given a specific relationship(s) is calculated following the methods described in Thompson (1975), Ge et al. (2010), and Ge et al. (2011). This method relies on 1) Identity by Descent (IBD) Coefficients (Table 1) and 2) joint genotype probabilities given IBD (Table 2).

**TABLE 1:** Identity by Descent (IBD) Coefficients derived from Li and Sacks (1954).

Relationship	φ_2_	φ_1_	φ_0_
Identical twins	1	0	0
Parent-child	0	1	0
Full sibs	¼	½	¼
Half sibs	0	½	½
1st cousins	0	¼	¾
Unrelated	0	0	1

**TABLE 2:** Joint genotype probabilities for two genotypes X and Y given IBD (i.e., φ).

Ordered genotypes (X, Y)	Joint probabilities: Pr(X,Y| φ)		
	IBD = 2 (φ_2_)	IBD = 1 (φ_1_)	IBD = 0 (φ_0_)
A_i_A_i_, A_i_A_i_	*p*_*i*_^*2*^	*p*_*i*_^*3*^	*p*_*i*_^*4*^
A_i_A_i_, A_j_A_j_	0	0	*p*_*i*_^*2*^ *p*_*j*_^*2*^
A_i_A_i_, A_i_A_j_	0	*p*_*i*_^*2*^ *p*_*j*_	2*p*_*i*_^*3*^ *p*_*j*_
A_i_A_j_, A_i_A_i_	0	*p*_*i*_^*2*^ *p*_*j*_	2*p*_*i*_^*3*^ *p*_*j*_
A_i_A_i_, A_j_A_k_	0	0	2*p*_*i*_^*2*^ *p*_*j*_ *p*_*k*_
A_j_A_k_, A_i_A_i_	0	0	2*p*_*i*_^*2*^ *p*_*j*_ *p*_*k*_
A_i_A_j_, A_i_A_j_	2*p*_*i*_ *p*_*j*_	*p*_*i*_ *p*_*j*_ (*p*_*i*_ + *p*_*j*_)	4*p*_*i*_^*2*^ *p*_*j*_^*2*^
A_i_A_j_, A_i_A_k_	0	*p*_*i*_ *p*_*j*_ *p*_*k*_	4*p*_*i*_^*2*^ *p*_*j*_ *p*_*k*_
A_i_A_j_, A_k_A_l_	0	0	4*p*_*i*_ *p*_*j*_ *p*_*k*_ *p*_*l*_

A_*i*_, *A*_*j*_, *A*_*k*_ and *A*_*l*_ are alleles at the locus with allele frequencies *p*_*i*_, *p*_*j*_, *p*_*k*_, and *p*_*l*_, respectively.

The likelihoods of the observed genotypes given two mutually exclusive hypotheses are compared. The likelihoods of the genetic data under each hypothesis are calculated as follows,

$$\mathrm{Pr}\left(X,Y\left|R)=\mathrm{Pr}(X,Y\right|\varphi 0\right)\times \varphi 0+\mathrm{Pr}\left(X,Y\left|\varphi 1)\times \varphi 1+\mathrm{Pr}(X,Y\right|\varphi 2\right)\times \varphi 2$$

where X and Y are two genotypes at a single locus, R is the hypothesized relationship, φ0, φ1, and φ2 are IBD = 0, 1, and 2, respectively, and Pr(X,Y|R) is the likelihood of the genotypes given R. The joint genotype probabilities of X and Y given IBD are shown in Table 2 (Ge et al., 2010; Ge et al., 2011). The cumulative likelihoods across multiple loci are the product of the likelihoods of each locus, assuming the loci are independent.

To avoid zero likelihoods due to mutations, a simple mutation model was implemented for the parent-child relationship. In this model, a constant mutation rate is set for any nucleotide difference; transitions (A↔G or C↔T) and transversions (A↔C, A↔T, G↔C, or G↔T) are treated as equally probable. Since the sequencing error rate likely is much higher than the mutation rate (e.g., 10^−8^), the default mutation rate is set at 10^−3^ (based on Q30 filtering, although it can be configured). While a mutation rate parameter is used herein, one could consider this rate as a genotyping error parameter of which the mutation rate is subsumed. This mutation model was applied only for the parent-child relationship. The impact of mutation for other relationships was considered negligible due to zero probability of IBD = 0. With the mutation model, the likelihood of a pair of genotypes given a parent-child relationship would be the product of the parent and the transmission probability from parent to child (the details can be found in Equation 6 in Ge et al. (2010).

### Software

KinSNP-LR was implemented in Python (version 3.10+). Input genotype data of tested individuals for KinSNP-LR were formatted as a tab-separated text format. Configurable parameters, such as MAF, MGD, mutation rate, genomic build (GRCh38 or GRCh37), and populations allowed for the exploration of a broad parameter space. KinSNP-LR outputs two files: a file contains the cumulative likelihoods and LRs for each population selected, and a file contains the details about each marker and the likelihoods for each population and relationship.

## Results

### Empirical genomic data

**FIGURE 2:**
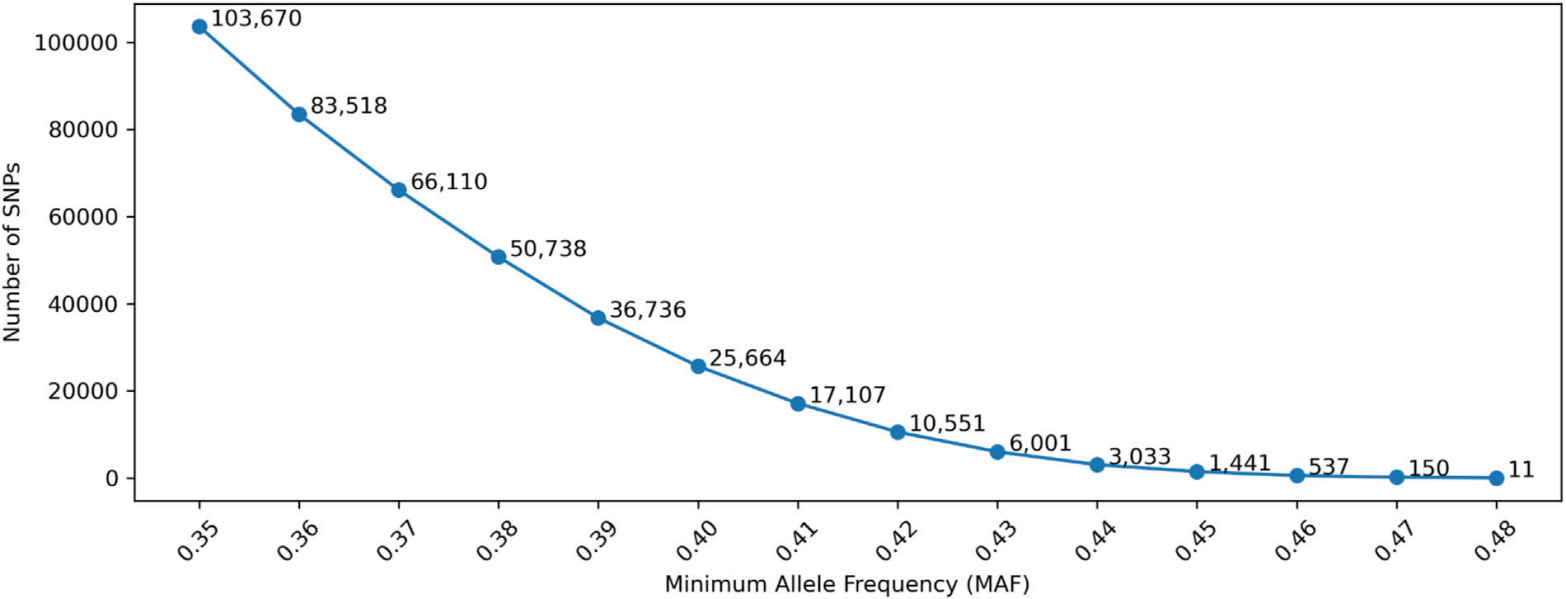
Number of selected SNPs with Minor Allele Frequency (MAF). Minimum Genetic Distance is not considered in this Figure.

The accuracy of traditional LR-based kinship analysis is directly dependent on the information content of each SNP and the total number of SNPs measured. To maximize information content of a SNP panel, a higher MAF is necessary. Figure 2 shows the number of selected SNPs with MAF thresholds, in which the allele frequencies in gnomAD were used. Only SNPs that had minor allele frequencies higher than a MAF threshold in each of the five populations (AFR, AMR, EAS, SAS, and NFE) were selected. With the MAF thresholds of 0.4 and 0.45, only 25,664 and 1,441 SNPs remained, respectively, which are appropriate for the threshold range for SNP selections.

In the 1,000 genomes data, there were 1,200 parent-child, 12 full-sibling, and 32 second degree pairs. In addition, 1,000 unrelated pairs were randomly selected with 200 in each of the five populations. In total, 2,244 pairs were selected to validate the LR method. For each pair, the likelihoods are calculated given each of the four relationships (unrelated, parent-child, sibling, and second degree), and each of the five populations were calculated. Assuming the population is known, the relationship is determined as the one with the maximum likelihood in that population.

**FIGURE 3:**
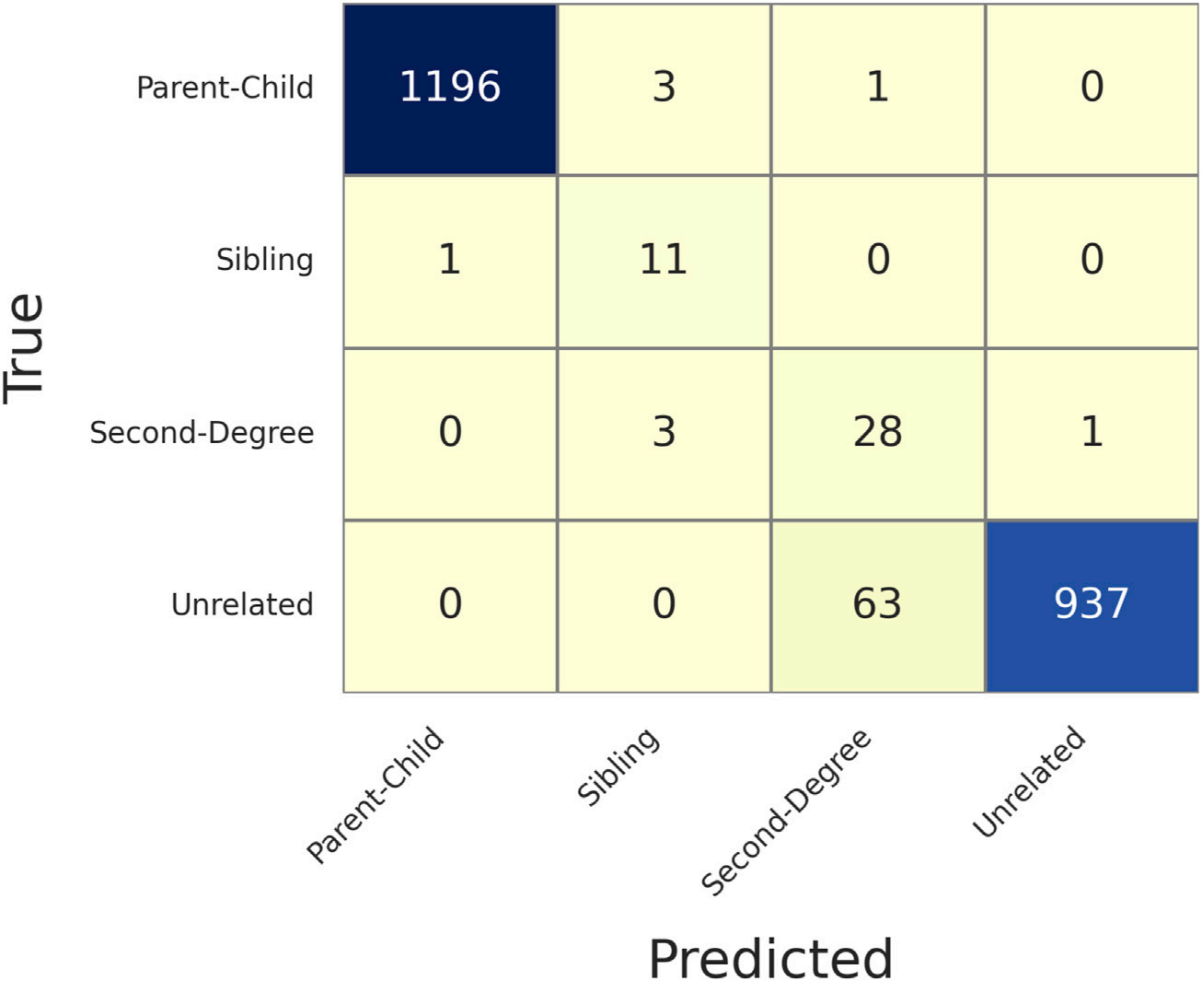
Confusion matrix of determining relationships given Minor Allele Frequency (MAF) = 0.4 and Minimum Genetic Distance (MGD) = 30 cM, with 126 selected SNPs. The rows are the true relationships, and the columns are the predicted relationships.

Figure 3 shows the confusion matrix of determining relationships given MAF = 0.4 and MGD = 30 cM with 126 SNPs selected. Based on this confusion matrix, the accuracy and the weighted F1 score could be calculated as 0.9679 and 0.9751, respectively, which were reasonably high given the limited number of SNPs available with the selection criteria described. As expected, most of the false associations were either with the first degree relationship (parent-child and sibling) or between unrelated and second degree.

Supplementary Table S1 gives more details of each of the false identifications in Figure 3. All the truly related but falsely identified pairs had the first-to-second LR less than 10, except HG00578-HG00582 (i.e., a ratio of 85), and many of them are about 1 or 2. But HG00578-HG00582 was correctly determined with lower MGD thresholds (and more SNPs), such as 10 cM (346 SNPs) and 20 cM (184 SNPs). The pairs truly unrelated but incorrectly identified as second degree all had first-to-second LRs less than 60, and 77.78% of them had ratios less than 10. Thus, the LR difference could be easily overcome by adding more SNPs. With a lower MGD of 10 cM, most of the errors were eliminated (Supplementary Table S2), except a few unrelated pairs were incorrectly determined as second degree. This observation is reasonable as many of the unrelated pairs in the 1,000 genomes data are not truly unrelated but distantly related to a certain degree (Fedorova et al., 2016), which resembles many actual relationship testing cases.

**FIGURE 4:**
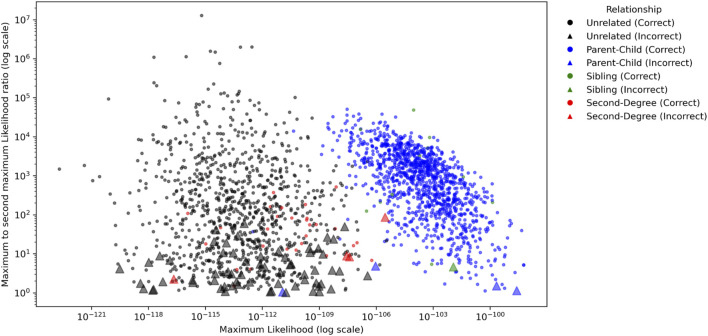
The ratios of the maximum likelihood and the second maximum likelihood among the populations for each pair for Minimum Genetic Distance (MGD) = 30 cM and Minor Allele Frequency (MAF) = 0.4. The circles and triangles represent the pairs with correct and incorrect identifications, respectively.

The ratio of the maximum and second maximum likelihoods among the populations were compared to investigate how confident a conclusion may be made to determine a relationship. As shown in Figure 4, in which MGD = 30 cM and MAF = 0.4 were applied, the parent-child and sibling relationships usually had several magnitudes higher likelihoods than the unrelated and second degree relationships. For parent-child and sibling, 53 out of 1,212 pairs (4.37%) had ratios less than 10, and 230 out of 1,212 pairs (18.98%) had ratios less than 100. For unrelated and second degree, 250 out of 1,032 pairs (24.22%) had the ratio less than 10, and 524 out of 1,032 pairs (50.78%) had ratios less than 100. In addition, all incorrectly identified pairs had the ratios less than 100, and 79.17% (15/72) of them were less than 10. Thus, with these 1,000 genomes data, a conclusion might be made with high confidence if the maximum likelihood of a relationship is 100 times greater than the second maximum likelihood.

**FIGURE 5:**
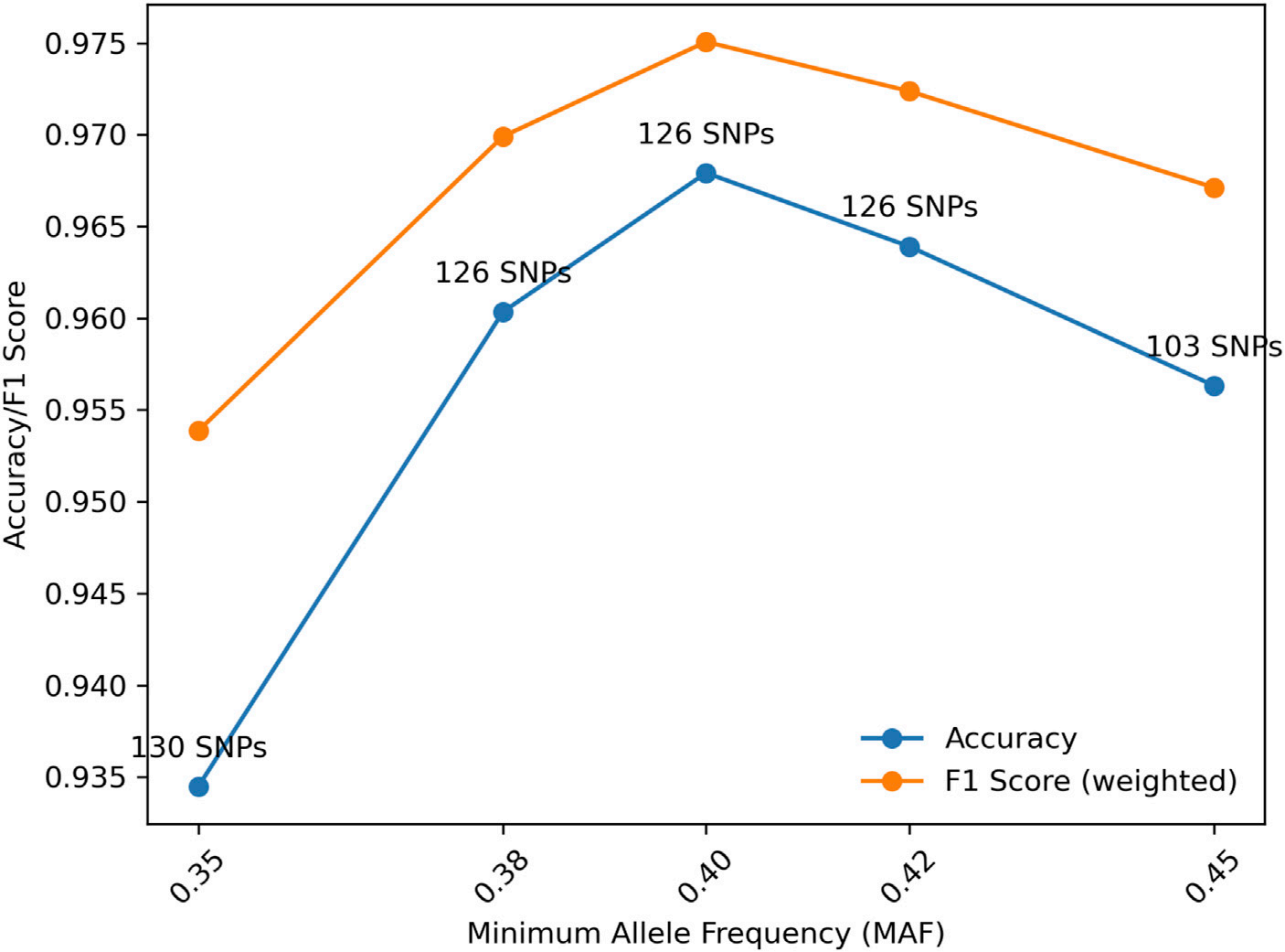
Distributions of accuracy and F1 score (weighted) with Minor Allele Frequency (MAF) given Minimum Genetic Distance (MGD) = 30 cM.

Further, for a fixed MGD = 30 cM, the effect of MAF also was investigated. As shown in Figure 5, the highest accuracy was reached with MAF = 0.4. With MAF = 0.35, more SNPs (i.e., 130) were selected but with the accuracy decreased, because more SNPs with lower information content were selected. MAF = 0.38, 0.4, and 0.42 all had the same number of SNPs selected but with different sets of SNPs. MAF = 0.4 had a higher accuracy than MAF = 0.38 probably because more informative SNPs were selected. MAF = 0.42 had a lower accuracy compared with MAF = 0.4, probably due to the randomness in the selection process. In general, a reasonable MAF threshold may be between 0.4 and 0.42 based on the current marker selection algorithm.

Table 3 displays the number of selected SNPs, the accuracies and weighted F1 scores of relationship identification given various MAF and MGD. The results were consistent with Figure 5 that MAF = 0.45 could lead to smaller numbers of selected SNPs and lower accuracies of relationship identifications with the current SNP selection method. As expected, lower MAFs and MGDs led to higher number of selected SNPs and higher relationship identification accuracies. With MAF = 0.4 and MGD = 10 cM, 346 SNPs were selected, and the accuracies and F1 scores were close to 100%. However, with MGD less than 50 cM, the selected SNPs may be considered linked to some degree. In practice, the linkage of pairs of markers separated by 30 or 40 cM may be sufficiently small to have little effect on the match probabilities or likelihood calculations (Buckleton and Triggs, 2006; Li et al., 2021). However, closer SNPs with MGDs of 10 or 20 cM may have substantial linkage effects on marker independence.

**TABLE 3:** The numbers of selected SNPs, accuracies, and weighted F1 scores given various Minor Allele Frequency (MAF) and Minimum Genetic Distance (MGD).

MGD	MAF					
	Number of selected SNPs		Accuracy		F1 score (weighted)	
	0.40	0.45	0.40	0.45	0.40	0.45
10 cM	346	211	0.9955	0.9799	0.9958	0.9831
20 cM	184	140	0.9733	0.9541	0.9792	0.9661
30 cM	126	103	0.9679	0.9563	0.9751	0.9671
40 cM	99	85	0.9407	0.9256	0.9573	0.9477
50 cM	80	71	0.9305	0.9100	0.9509	0.9382

### Simulation data

**FIGURE 6:**
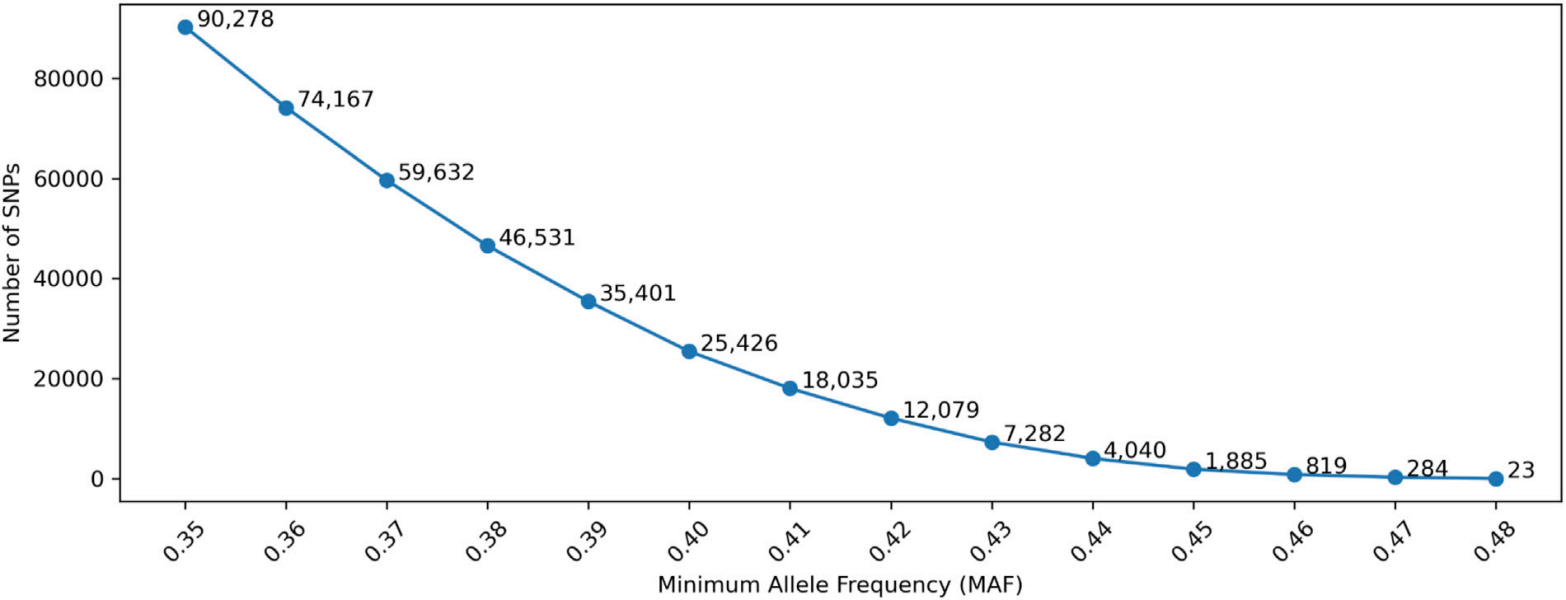
Number of selected SNPs with Minor Allele Frequency (MAF). Minimum Genetic Distance is not considered in this Figure.

Similar studies were conducted with simulation data. The number of SNPs after MAF filtering (as shown in Figure 6) were very similar to the numbers in Figure 2 (i.e., empirical data), in which the allele frequencies in the 1,000 genomes data were used. The differences were due to the slight allele frequency differences between gnomAD and the 1,000 genomes data.

**FIGURE 7:**
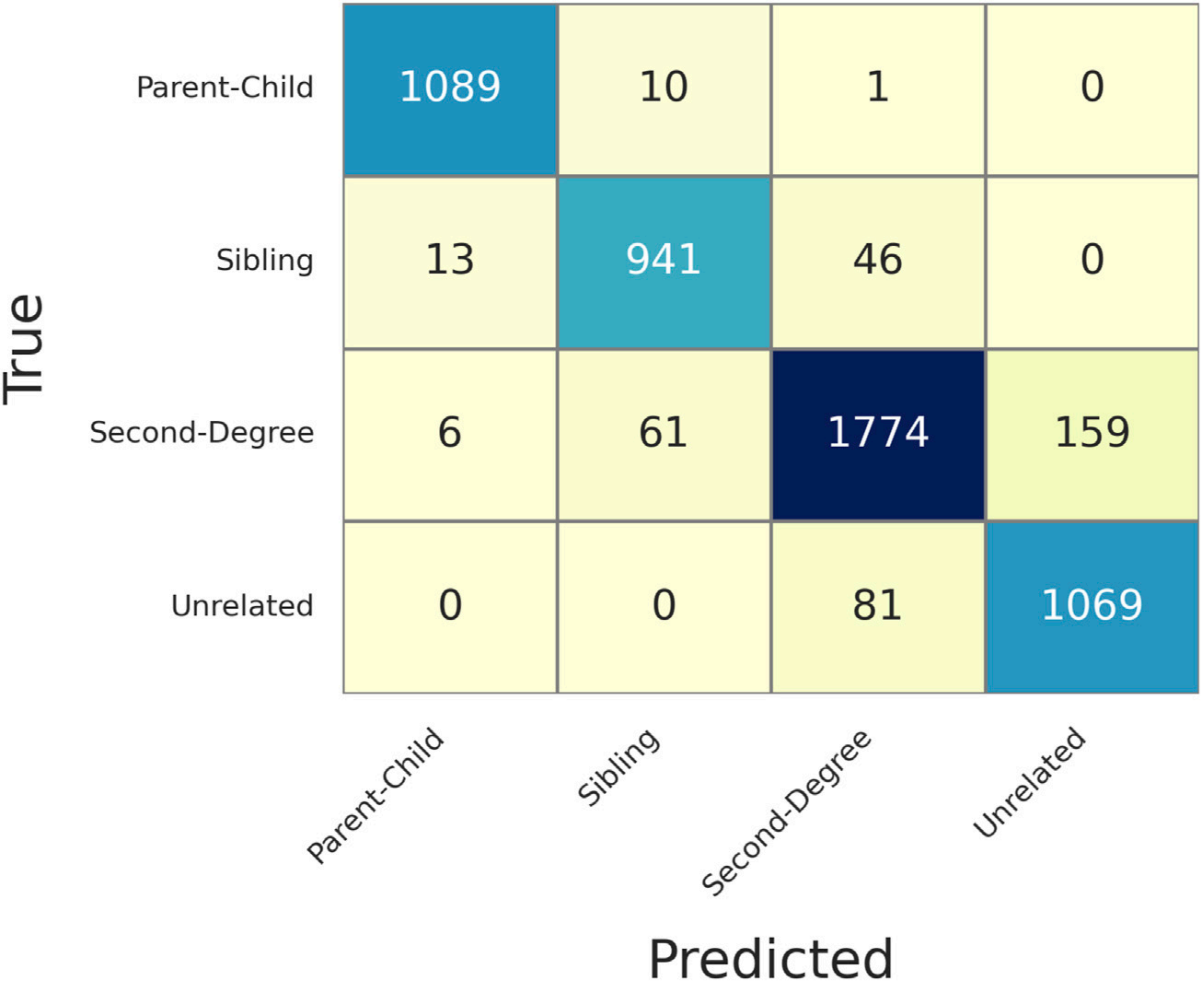
Confusion matrix of determining relationships given a Minor Allele Frequency (MAF = 0.4) and Minimum Genetic Distance (MGD) = 30 cM, with 130 selected SNPs. The rows are the true relationships, and the columns are the predicted relationships.

Figure 7 shows the confusion matrix of determining relationships given MAF = 0.4 and MGD = 30 cM with 130 SNPs selected. Based on this confusion matrix, the accuracy and the weighted F1 score were both 0.9282, which were slightly lower than those with empirical data. This observation is likely due to the simulated data having more sibling and second degree relationships and that more distant relationships usually have lower accuracies in testing. The confusion matrices of ASW, CHB, and MXL can be found in the Supplementary Material C.

Supplementary Table S3 gives more details of each of the false identifications in Figure 7. 97.1% (i.e., 366/377) of the pairs were falsely identified with a first-to-second LR ratio greater than 100, and 82.5% (311/377) of ratios were less than 10. Like the empirical data, when MGD reduces to 10 cM (Supplementary Table S4), the accuracy substantially increases due to more SNPs being selected.

**FIGURE 8:**
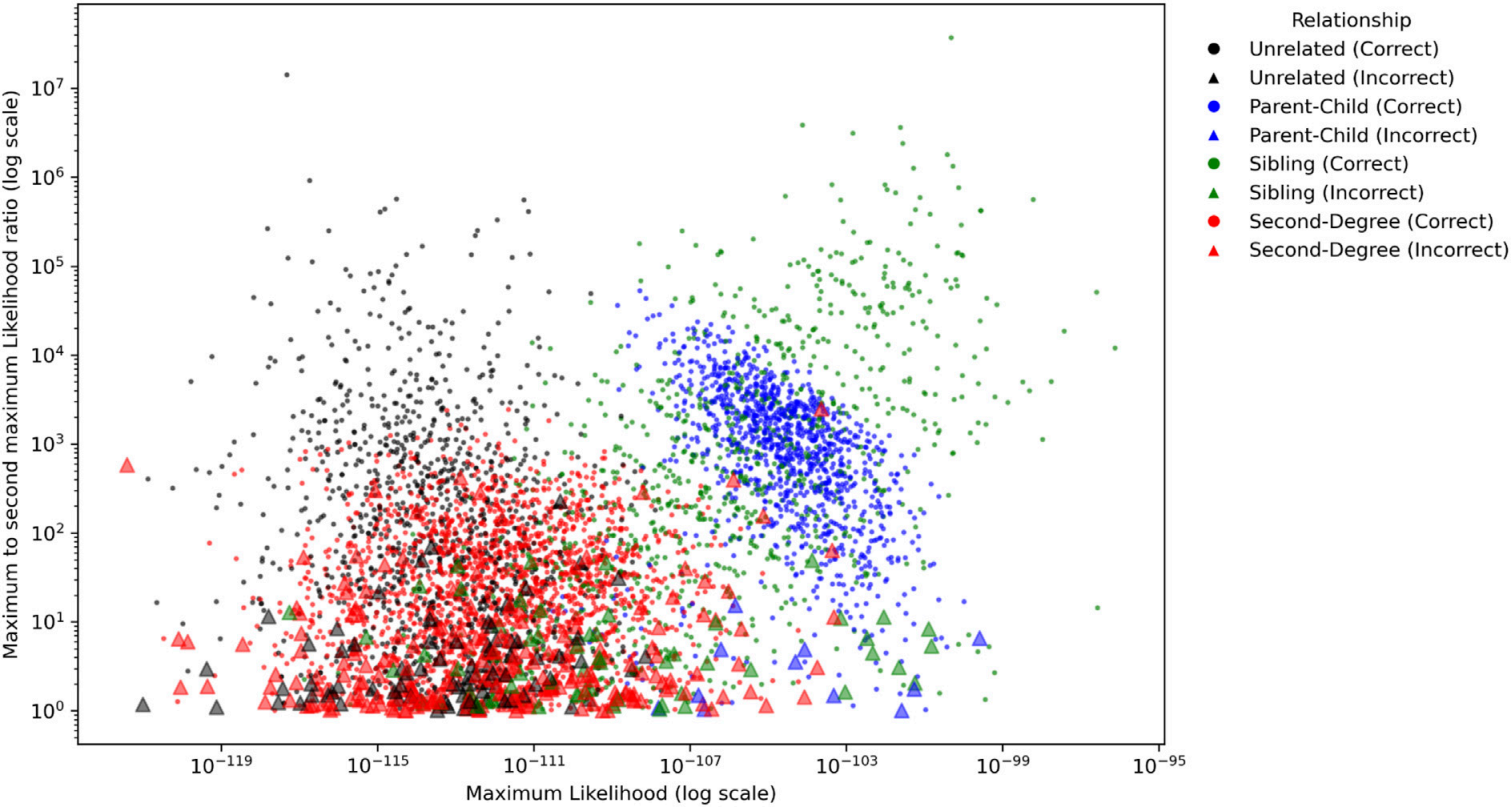
The ratios of the maximum likelihood and the second maximum likelihood among the populations for each pair for Minimum Genetic Distance (MGD) = 30 cM and Minor Allele Frequency (MAF) = 0.4. The circles and triangles represent the pairs with correct and incorrect identifications, respectively.

The ratio of the maximum and second maximum likelihoods for the CEU population with MGD = 30 cM and MAF = 0.4 were plotted in Figure 8. Like the results with empirical data, the parent-child and sibling relationships usually had several magnitudes higher likelihoods than the unrelated and second degree relationships. For parent-child and sibling, 226 out of 2,100 pairs (10.76%) had ratios less than 10, and 554 out of 2,100 pairs (26.38%) had ratios less than 100. For unrelated and second degree, 971 out of 3,150 pairs (30.86%) had the ratio less than 10, and 2,200 out of 3,150 pairs (70.48%) had ratios less than 100.

In addition, 82.49% (311/377) of the incorrectly identified pairs had ratios less than 10, 97.08% (366/377) of them were less than 100, and only 1 out of 377 had the ratio >1,000. Thus, within unrelated and these tested close relationships, it is highly confident to conclude a relationship if the maximum likelihood of a relationship is 1,000 times greater than the second maximum likelihood. Otherwise, an inconclusive interpretation could be made.

The effect of MAF also was investigated with the simulation data. The peak accuracy was obtained with MAF = 0.3, which is slightly different from the distributions generated from empirical data, likely due to the different allele frequencies in the gnomAD and the 1,000 genomes data. The accuracy difference across different MAFs may not be substantial, and the distribution is rather random due to the availability of the SNPs and allele frequencies used in analysis. For samples from real cases, the SNP profiles generated may have a much smaller number of SNPs with high quality. Thus, multiple MAFs between 0.3 and 0.4 may be tried to achieve the highest likelihood and/or LR.

**FIGURE 9:**
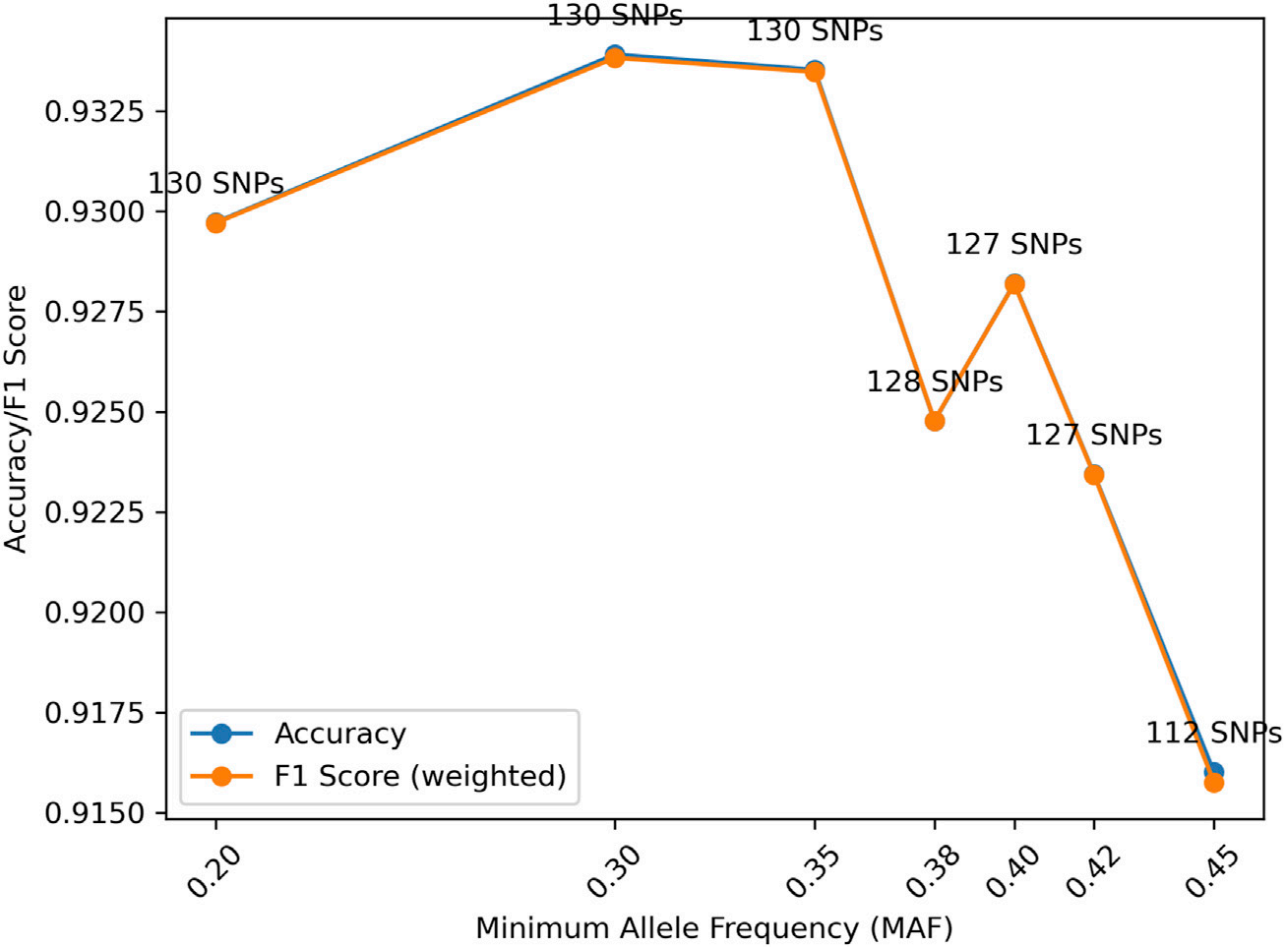
Distributions of accuracy and F1 score (weighted) with Minor Allele Frequency (MAF) given Minimum Genetic Distance (MGD) = 30 cM. The accuracy and F1 score (weighted) are almost identical due to the relatively even numbers of samples for each relationship.

It is worth noting that the gnomAD panel was used to filter the SNPs in the 1,000 genomes data in the simulation study. However, many SNPs in the gnomAD panel have lower MAF than 0.3 than in the 1,000 genomes data. Because of this difference, MAF = 0.2 is in Figure 9.

The effect of genotyping errors also was evaluated. LRs with various genotyping error rates in simulation and in LR calculation were calculated, and the accuracies and F1 Scores (weighted) were summarized in Table 4 (the confusion matrices can be found in Supplementary Material D). Apparently, lower error rate increases the accuracy. An error rate of 0.01 may still be acceptable for LR calculations (i.e., the accuracy is reduced from 0.9282 to 0.9198 with error rates increasing from 0.001 to 0.01). Error rate in LR calculations also is important. With 0.05 simulation error, LR calculations with the same error rate (i.e., 0.05) can substantially increase the relationship test accuracy (i.e., 0.8663 and 0.7907 with 0.05 and 0.01 LR calculation errors, respectively). In general, low genotyping error is extremely important for LR based relationship testing. It is necessary to filter out low quality variants with certain measures (such as read depth, variant quality scores, etc.) before calculating LRs.

**TABLE 4:** The accuracies and F1 scores (weighted) for various simulation genotyping error rates and LR calculation error rates.

Simulation error	LR calculation error	Accuracy	F1 score (weighted)
0.001	0.001	0.9282	0.9282
0.01	0.001	0.9110	0.9114
0.01	0.01	0.9198	0.9198
0.05	0.001	0.7907	0.7805
0.05	0.05	0.8663	0.8660

CEU population, Minor Allele Frequency (MAF = 0.4), and Minimum Genetic Distance (MGD) = 30 cM were used.

## Discussion and conclusion

This paper describes the development and validation of a LR-based methodology designed to conduct close kinship relationship testing by dynamically selecting informative SNPs based on MAF and MGD from genome-wide SNP data. Our approach is unique in that it integrates dynamic SNP selection based on output data, rather than relying on a fixed, pre-selected SNP set. The described approach bridges modern genomic datasets with the traditional LR-based framework, allowing kinship testing laboratories to leverage comprehensive genomic information while maintaining established LR-based interpretive methodologies. KinSNP-LR, as implemented in this study, does not incorporate comprehensive population parameters, such as Fst or linkage between markers; these are features that can be considered in future iterations. This initial implementation is intended to demonstrate the core logic and workflow of dynamically integrating SNP selection with LR calculations for close kinship analysis. The system is evaluated for accuracy using empirical and simulated datasets.

However, the traditional LR framework assumes marker independence, necessitating the selection of only a limited subset of SNPs from the total typed data to minimize the confounding effects of linkage and LD^31^. The current implementation of the methodology supports parent-child, full-sibling, and second degree relationships, as well as distinguishing unrelated individuals. These close relationships are most commonly encountered in missing and unidentified persons cases handled by medical examiner offices, where the goal is to confirm identity rather than generate investigative leads. While more distant relationships could be incorporated in future iterations by including linked SNPs, such comparisons generally are less fruitful for identity confirmation due to the higher number of potential matches and reduced specificity.

This LR methodology assumes access to high-quality genotyping data. When genotypes are derived from low-quality or low-quantity DNA samples, rigorous quality control measures are necessary to ensure accurate relationship testing, which may include the use of metrics such as genotype quality scores, read depth, or other laboratory-specific thresholds for SNP quality. The default mutation rate in the methodology is set at 0.001, corresponding to the Q30 threshold, as genotyping errors are significantly more frequent than the actual biological mutation rate (∼10^−8^). For datasets of lower quality, the mutation rate can be adjusted to higher values (e.g., 0.01) to account for increased error rates. In this study, the mutation rate (or better stated typing error rate) was performed only on parent-child relationships as is typically done in such cases. However, one could consider applying the typing error rate to all relationships tested herein since typing error will impact all sequence data.

Additionally, targeted panels may be considered, as they can be enriched to increase sensitivity of detection. The LR approach would remain similar, though the number of candidate SNPs will be fewer unless the DNA is highly degraded. For close relationships up to second degree, the number of SNPs may be sufficient, as only a limited number are needed. However, enrichment approaches still may suffer from differential detection of SNP states, and overall data quality will impact success. While the choice between targeted panels and WGS has historically been influenced by cost and laboratory preference, declining sequencing costs are making WGS increasingly attractive due to its broader genomic coverage and greater utility in distant kinship inference. This LR tool was developed to accommodate WGS data, but if one would like to use it with a targeted panel, the panel should be evaluated for its ability to include a maximum number of unlinked (or loosely linked) SNPs.

Substructure, which likely exists at some level, was not included in this iteration of the software because these SNPs likely exhibit low Fst values, particularly for each individual population. The impact of Fst on calculations involving high frequency SNP alleles should minimize their impact of substructure on LR calculations and support robust relationship testing across populations. Future versions of KinSNP-LR could explore the impact of a Fst correction based as described by Balding and Nichols (1994).

The selection of an appropriate large set of candidate SNPs for relationship testing was derived from a preselected gnomAD panel containing 222,366 SNPs. These SNPs were filtered based on a MAF threshold of 0.3. Subsequently, the data were further subset to include SNPs located in genomic regions identified by Genome-in-a-Bottle as being easy to sequence or genotype. One might consider a much lower MAF, such 0.01, as rare alleles may increase the LR; however, such SNPs would not apply to all kinship comparisons as they are quite uncommon and have little value in most cases. A high MAF ensures that the SNPs from the large, preselected candidate panel of 222,366 SNPs would be applicable routinely. The high MAF threshold enhances the informativeness of selected SNPs, while reducing risk of unintended medical interpretations. This filtering strategy ensures a robust and unbiased dataset, providing a solid foundation for FGG as well as for other forensic applications, such as mixture interpretation. These selection steps provided SNPs that are amenable to validation studies performed herein, supporting reliability and reproducibility in forensic applications.

There have been reports by Mostad et al. (2023) and Andersen et al. (2025) that also provide approaches to use LRs with kinship analyses based on SNP data. They both have some positive features and limitations, as does KinSNP-LR. Mostad et al. (2023) use the Lander-Green algorithm, basically a Hidden Markov model (HMM), like the methods described by Epstein et al. (2000) and Boehnke and Cox (1997). Their method can exploit larger amounts of SNP data with the assumption of linkage equilibrium and no genetic interference, in which the likelihood of individuals given a defined relationship is calculated using the allele frequencies, the recombination fractions between the markers, and transition probabilities of the IBD values of linked markers. Thus, instead of using a maximum of a few hundred SNPs with the assumption of little or no linkage between the markers, HMM-based methods in theory could use all available SNPs to increase the accuracy of the relationship tests. However, the Mostad et al. (2023) approach does not address mutation or genotyping error which could be a problem with low quality data, although mutation rates could be added to their model. The benefits of HMM may be limited for close relationships, as shown herein and the accuracy is already very high for close relationships with a few hundred SNPs. However, if LRs are desired for more distant relationships, HMM-based methods can be utilized in subsequent versions of KinSNP-LR. Andersen et al. (2025) used a preselected panel of 43 SNPs with relatively high MAF for LR calculations accounting for genotype errors for direct comparison of single source samples to persons of interest. Our approach is in some ways similar to that of Andersen et al. (2025) but extended to indirect kinship comparisons and makes use of more SNPs. KinSNP-LR provides a model for possible solutions to address LR/SNP-based calculations by dynamically selecting high MAF SNPs common to several population groups, MGD to account for linkage effects, incorporation of a basic mutation (or genotyping error) rate model, and being flexible on a case-by-case perspective.

In addition, including multiple reference family members instead of pairwise comparisons (i.e., joint probabilities), which are not described herein because the current genetic genealogy applications are mostly with pairwise relationship tests, can be considered in future studies. For cases with more than one family reference sample, a pedigree LR can be calculated capturing all genetic information from all the references. Ge et al. (2010), based on the Elston-Stewart algorithm (Elston and Stewart, 1971), provide the methods for calculating a pedigree likelihood which incorporated genetic mutation, population substructure and accommodations for missing genetic data.

There are other ways to implement algorithms other than the one used herein which is a traditional, case-specific approach for LR calculation based on genotype combinations, where the formulae differ according to the genotype combination of the participants. For example, unified LR calculation formulae could be considered to address all genotype combinations with a single, more generalizable equation (Egeland, et al., 2017; Ma, et al., 2024). This would reduce reliance on complex code with numerous conditional statements which in turn potentially could reduce computational efficiency and increase risk of implementation errors, especially when dealing with high-density SNP data. KinSNP-LR is designed for close relationship testing for a specific targeted audience, such as medical examiners. The code for this software is only a few hundred lines. Therefore, these concerns that can be obviated to some degree with a unified LR calculation formulation are less likely an issue with KinSNP-LR which has been tested under the conditions shown in this study. In future iterations, where more distant and complex relationships would be addressed, the unified approach will be considered.

The validation results from both empirical and simulation data show high accuracies of close relationships, particularly for first degree relationships. One potential limitation is that the sample size of 50 pedigrees may be considered small. However, the data shown in Figures 6–9 and Table 4 support that the sample size is sufficient to evaluate the performance of the software tool for the tested relationships. On another point, the ratios for selecting relationships with maximum and second maximum likelihoods differed for empirical data (>100) and simulated data (>1,000). Sampling is a potential explanation for the observed differences between empirical and simulation results. The empirical data have less samples, which tend to result in lower ratios overall. With more samples, as with the simulated data, larger ratios will be observed. These differences between the maximum and the second maximum likelihoods are merely observations and may not be construed as a recommended threshold(s) for casework. Threshold selection will be done at the laboratory level and based on the risk profile that a laboratory or jurisdiction defines operationally.

Comparisons with other kinship software were not conducted. The reasons were the input data format and requirements, or even assumptions and methods, are different for different software programs. To seamlessly integrate with WGS-based genetic genealogy application, KinSNP-LR can accept any VCF file without pre-defined panels, which is unique among all LR-based relationship testing software programs. Due to the different input data, assumptions, methods, and/or software implementations, different software programs may use different sets of variants, population features, and methods in relationship testing, which will lead to different LR results or even different conclusions but not provide insight into accuracy of any given software. Thus, the evaluation (e.g., accuracy) of any software program should be conducted with ground truth data, which is what was done in this study, instead of comparing with other software programs.

In addition, the WGS data generated for some forensic samples may yield 0.5X to 5X coverage. If, for example, DeepVariant (Poplin et al., 2018) were used to call variants with additional high-quality filters, such as Genotype Quality (GD) ≥ 30 (equivalent to 99.9% genotyping accuracy) and a minimum read depth ≥5, such samples may yield limited data. However, for higher quality samples, typically ∼1,600 to ∼350,000 high quality variants could be detected for relationship testing. Even with further MAF and MGD filtering, there should be enough variants for close relationship tests, which usually only require dozens to a few hundred SNPs. On the other hand, relationship test software programs that require pre-selected panels may not work well for WGS data generated from typical forensic samples, as there may not be enough SNPs recovered with smaller pre-selected SNP panels after stringent quality filtering. A pre-selected panel may start with upwards of ∼3,500 to ∼10,000 compared with millions or tens of millions of SNPs by WGS.

In summary, the methodology presented in this study marks a significant step forward in bridging traditional forensic relationship testing with modern genomic technologies. By applying a LR-based framework with dynamically selected high-MAF SNPs, forensic laboratories can harness the data generated by WGS while maintaining compliance with accredited relationship testing standards. The use of unlinked markers and case-specific SNP selection distinguishes this method from existing LR-based kinship tools, making it especially applicable for FGG and other applications requiring statistically robust assessments of close relationships. This approach is particularly important for medical examiners, who are focused on confirming identity, which is a process that typically relies on comparisons of close relatives. In cases where STR testing is not possible due to degraded or limited DNA, this SNP-based LR approach offers a reliable and scientifically defensible alternative. As the field evolves, these advancements provide a strong foundation for broader adoption of relationship testing approaches that combine the rigor of traditional statistical models with the scalability of modern genomics.

## Data Availability

The original contributions presented in the study are included in the article/Supplementary Material, further inquiries can be directed to the corresponding author.
