# Supplementary material for: A likelihood ratio framework for inferring close kinship from dynamically selected SNPs
Source: Front Genet. 2025 Jul 23;16:1635734. doi: 10.3389/fgene.2025.1635734 (PMC12325062; doi:10.3389/fgene.2025.1635734)

**Supplementary Material - A**

**A large, preselected SNP panel as data foundation for SNPLR**

The accuracy of LR-based kinship analysis is directly dependent on the selected SNPs and therefore several considerations influence the choices. Neighboring variation can make genotyping difficult on some platforms, so SNPs that are close to known indels were removed. Also, the ‘difficult regions’ identified during the Genome-In-a-Bottle (GIAB) 4 project were excluded. In order to be compatible with older datasets, and prepared for future ones, positions were removed if they were handled differently across different versions of the reference genome.

To maximize information content the chosen SNPs should have a high MAF (i.e., approaching 0.5) in a wide range of subpopulations. To identify such SNPs, population data in gnomAD v4 (Chen et al., 2024) were investigated. gnomAD v4 is based on GRCh38 and distributed in VCFv4.2 formatted files. The VCF format permits multiple representations of equivalent information. gnomAD uses a multiline VCF representation with only a single alternate allele per line. This format is well suited for capturing the alternate allele frequency and similar annotation in the INFO column; however, it necessitates looking at multiple lines to recognize multiallelic SNPs.

To select an initial set of SNPs, MAF was set at 0.3 based on the population data (n= 730,947 exomes and 76,215 whole genomes, all mapped to the GRCh38 reference sequence) in gnomAD v4 (<https://gnomad.broadinstitute.org/downloads>). These data were downloaded and filtered based on the following criteria:

- The VCF record must have a FILTER field with the value ‘PASS’.
- Records where the REF & ALT are not one nucleotide are considered Indels and any SNPs which are overlapped by their longest form were eliminated.
- SNPs must be on an autosome (chr1 - chr22) and biallelic.
- The MAF of 0.3 and must be inclusive globally in all gnomAD v4 populations listed herein.

The populations listed in gnomAD v4 are:

- Admixed American
- African
- Ashkenazi Jewish
- East Asian
- European (includes Finnish)
- Middle Eastern
- Remaining Individuals (includes Amish)
- South Asian

Variants were further filtered by using the intersect tool from pybedtools (Quinlan et al., 2010; Dale et al., 2011) to compare against the Genome In a Bottle (GIAB) (Wagner et al.. 2022) “not in all difficult regions” stratification bed file (<https://ftp-trace.ncbi.nlm.nih.gov/ReferenceSamples/giab/release/genome-stratifications/v3.3/GRCh38@all/Union/GRCh38_notinalldifficultregions.bed.gz>) (Olson et al., 2022) . This approach removed a variety of challenging regions including segmental duplications, difficult to map regions, homopolymers, tandem repeats, the major histocompatibility complex (MHC) regions, and regions of high/low GC content.

Each SNP then was mapped onto hg19/GRCh37 and T2T-CHM2.0 via pyliftover (<https://github.com/konstantint/pyliftover>) using the chain files:

- <https://hgdownload2.soe.ucsc.edu/goldenPath/hg38/liftOver/hg38ToHg19.over.chain.gz>
- <https://hgdownload.gi.ucsc.edu/hubs/GCA/009/914/755/GCA_009914755.4/liftOver/hg38-chm13v2.over.chain.gz>

A SNP was rejected if the liftover onto either of these other references failed to map to exactly one position, or the target chromosome was not one of the standard autosomes. This approach should ensure that the candidate SNP panel is well suited for legacy data, is compatible with GRCh38, leverages what has been learned from T2T-CHM13, and makes a reasonable effort for stability with likely future changes. The lifted results then were sorted into their respective correct ordering, and the final panel was suitable for all three reference genomes. Since a common source of inconvenience is the presence of a “chr” prefix on some sources (such as UCSC RefSeq) and the absence of this prefix on others (such as Ensembl) one final step used “bcftools annotate --rename-chrs” to prepare versions of the GRCh37 VCF with and without the prefix.

The filtered gnomAD data eventually included 222,366 SNPs, which serve as the data foundation for SNP selection in KinSNP-LR. Selecting SNPs for forensic testing with high MAF can mitigate privacy concerns because they likely have low predictive power, which ensures strong safety and security measures to protect genetic data.

**References:**

1. Chen S, Francioli LC, Goodrich JK, Collins RL, Kanai M, Wang Q, Alfoldi J et al. (2024) A genomic mutational constraint map using variation in 76,156 human genomes. Nature 625(7993):92-100.
2. Quinlan AR, Hall IM (2010) BEDTools: a flexible suite of utilities for comparing genomic features. Bioinformatics 26(6):841–842.
3. Dale RK, Pedersen BS, Quinlan AR (2011) Pybedtools: a flexible Python library for manipulating genomic datasets and annotations Bioinformatics 27(24):3423–3424.
4. Wagner J, Olson ND, Harris L, Khan Z, Farek J, Mahmoud M, Stankovic A, et al. (2022) Benchmarking challenging small variants with linked and long reads. Cell Genomics 2, 100128.
5. Olson ND, Wagner J, McDaniel J, Stephens SH, Westreich ST, Prasanna AG, Johnason E, et al. (2022) PrecisionFDA Truth Challenge V2: Calling variants from short and long reads in difficult-to-map regions. Cell Genomics 2(5):100129.

**Supplementary Material - B**

The pseudocode of dynamic SNP Selection under Minor-Allele‐Frequency and Minimum-Genetic-Distance Constraints. This dynamic SNP-selection algorithm applies a high minor-allele-frequency (MAF) filter to a large candidate panel, then sweeps each chromosome once from the end inward, greedily choosing the first marker and every subsequent marker that lies at least Δ centimorgans away from the last one. Because all retained SNPs have similarly high MAF, information content per marker is nearly uniform, so simply maximizing the count within the distance constraint maximizes cumulative power. Assuming the SNPs in the vcf file have been sorted, the procedure runs in linear time, yet it approximates the optimal distance-constrained subset-selection problem, which is NP-hard for exact solutions, closely enough for forensic and association applications.

The following are the notations used in the pseudocode.

S: Set of candidate SNP records ⟨ chromosome, genetic-position (cM), MAF ⟩

θ: Minor-allele-frequency threshold (e.g., 0.40)

Δ: Minimum genetic distance (MGD) between any two selected SNPs (e.g., 30 cM)

P: Dynamic panel of SNPs satisfying MAF ≥ θ and pairwise distance ≥ Δ within each chromosome, or the final panel

—————————————————————————————————————

Dynamic_SNP_Panel(S, θ, Δ)

1: P ← ∅ # Final panel
2: for each chromosome c in genome do
3: L_c ← { s ∈ S | s.chromosome = c ∧ s.MAF ≥ θ } # High-MAF filter
4: last_pos ← −∞ # No SNP yet selected
5: for each SNP s in L_c do # Single left-to-right sweep
6: if (s.position − last_pos) ≥ Δ then
7: P ← P ∪ { s } # Select SNP
8: last_pos ← s.position
9: end for
10: end for
11: return P

—————————————————————————————————————

Note: “#” is the comment notation.

**Supplementary Material - C**

Confusion matrices of ASW, CHB, and MXL with the simulation data. The accuracies and F1 scores (weighted) were similar to those of CEU.

ASW; 130 SNPs; Accuracy= 0.9286; F1 Score (weighted) = 0.9285


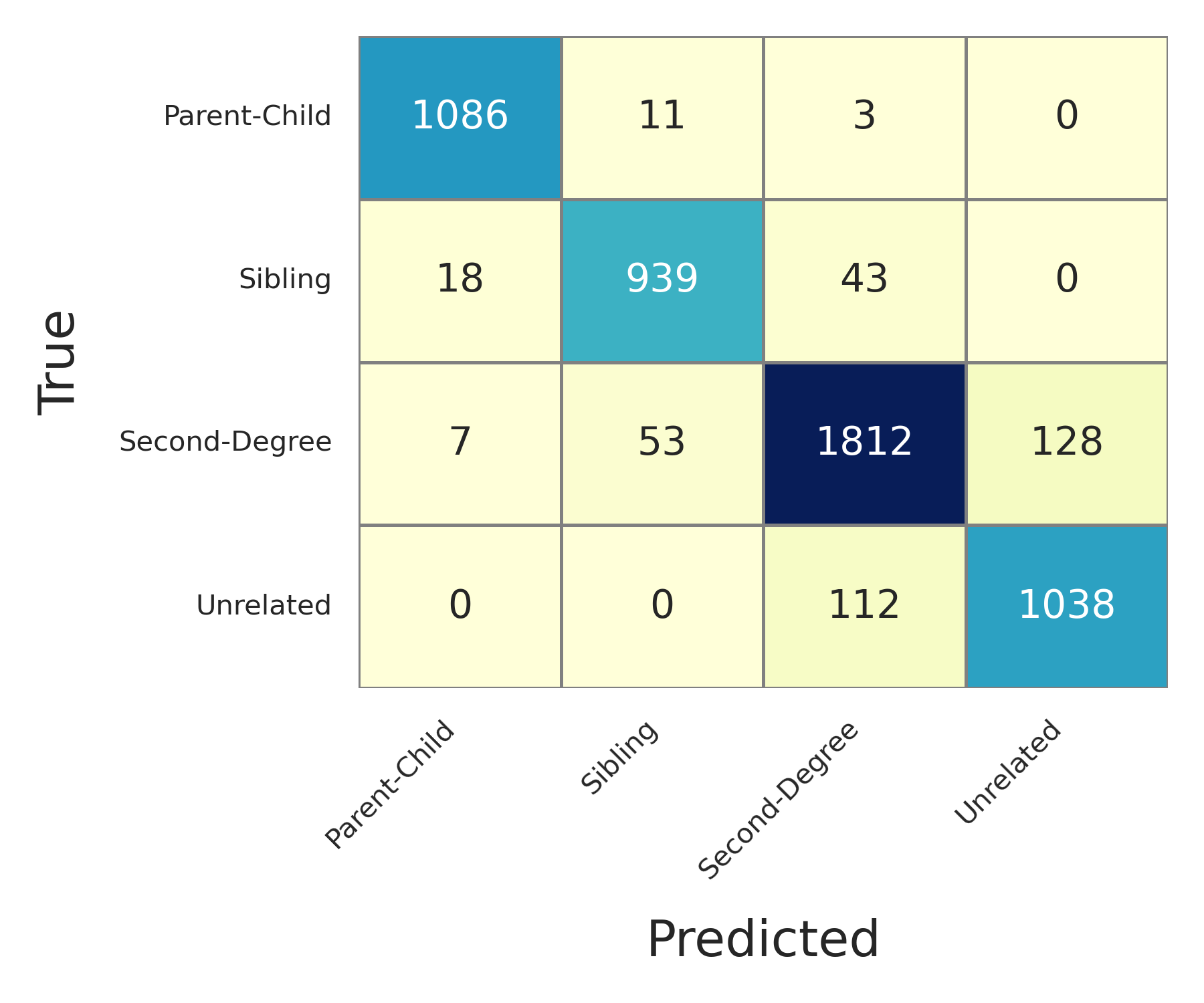


CHB; 130 SNPs; Accuracy= 0.9398; Weighted F1 Score = 0.9397


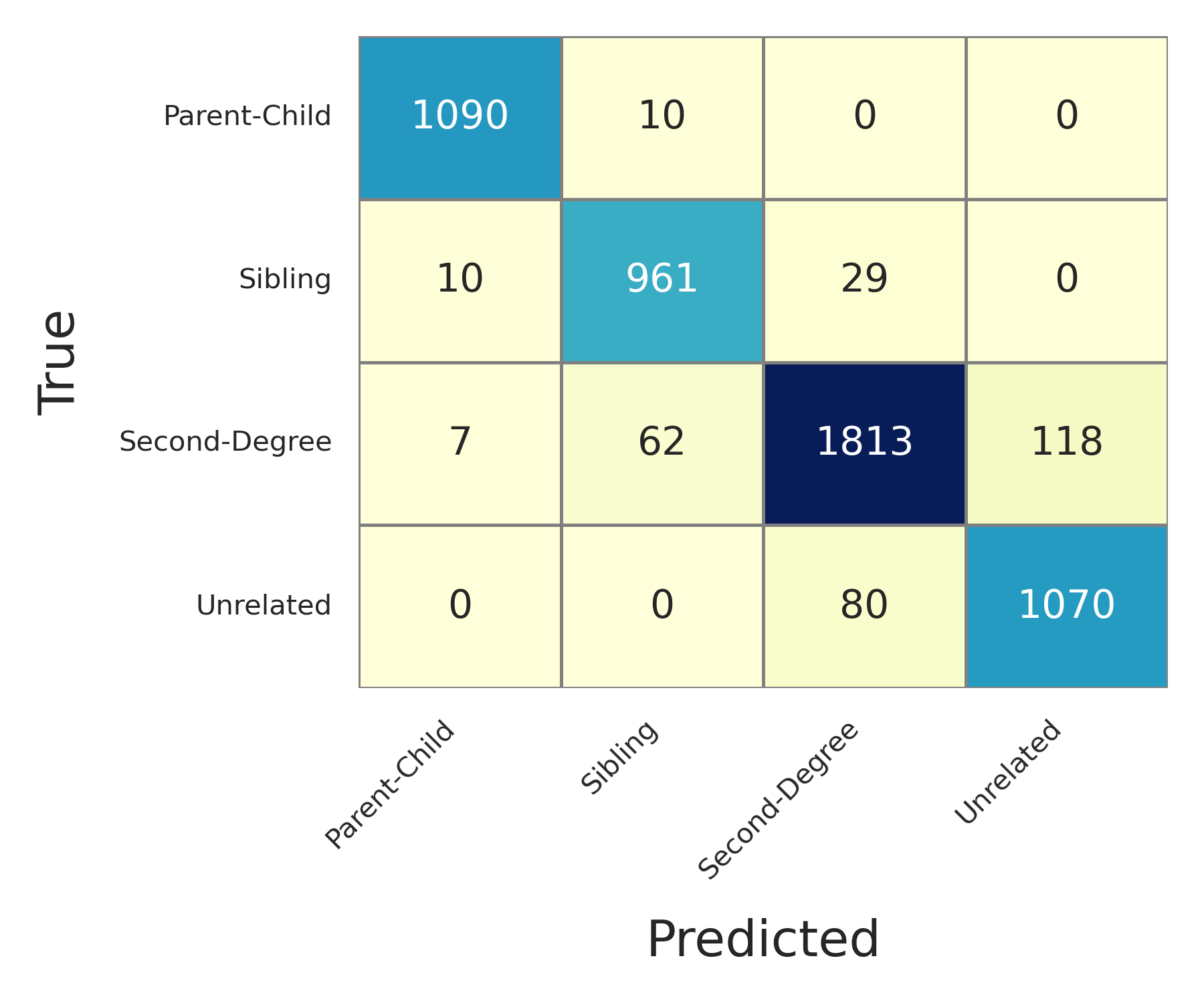


MXL; 130 SNPs; Accuracy= 0.9358; Weighted F1 Score = 0.9357


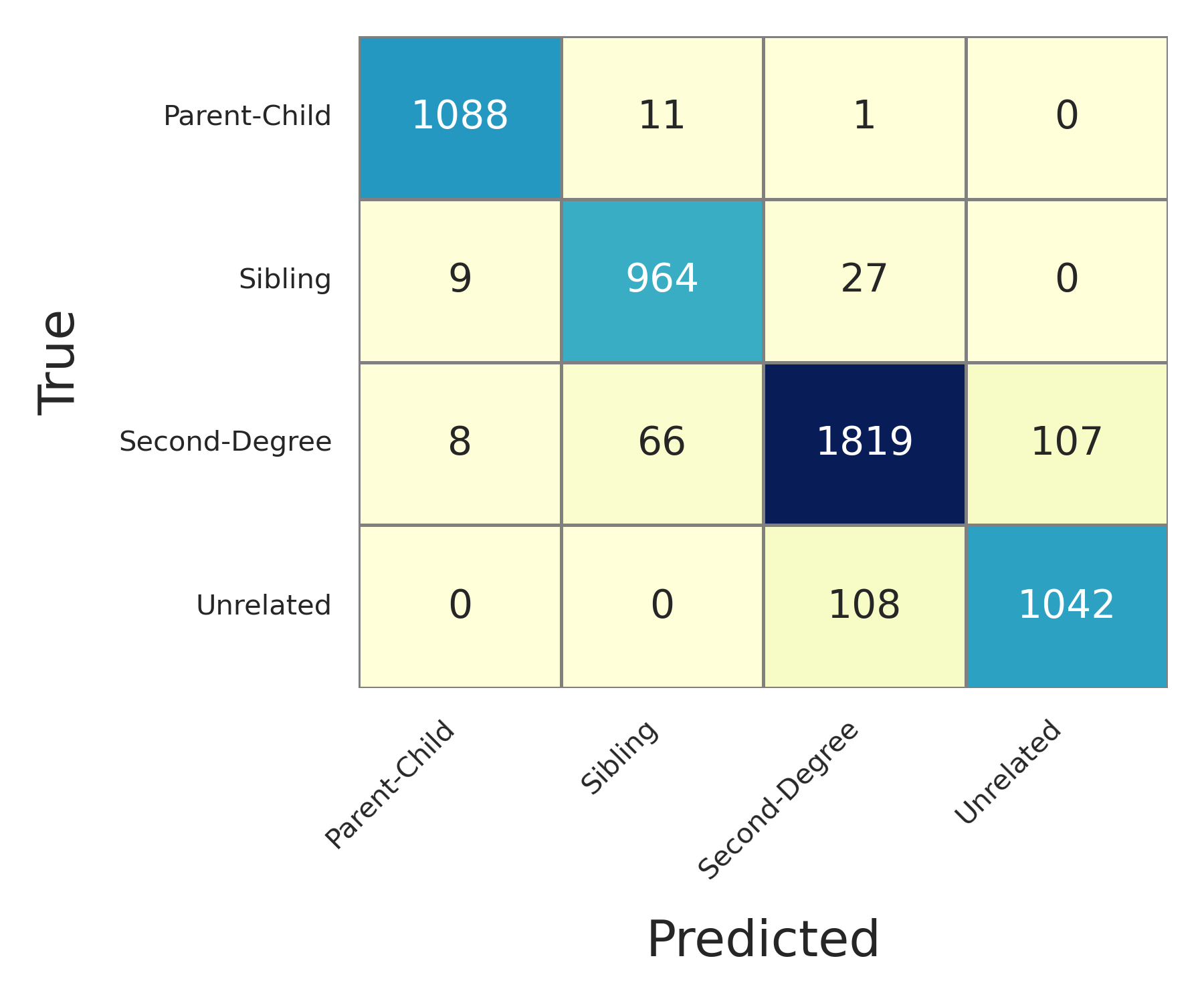


**Supplementary Material - D**

The confusion matrices, accuracies, and F1 scores (weighted) for various simulation genotyping error rates and LR calculation error rates. CEU population, MAF=0.4, and MGD=30cM were used.

CEU, MGD=30cM, MAF=0.4; simu error= 0.01; LR calculation error = 0.001

Accuracy = 0.9282; F1 Score (weighted) = 0.9282


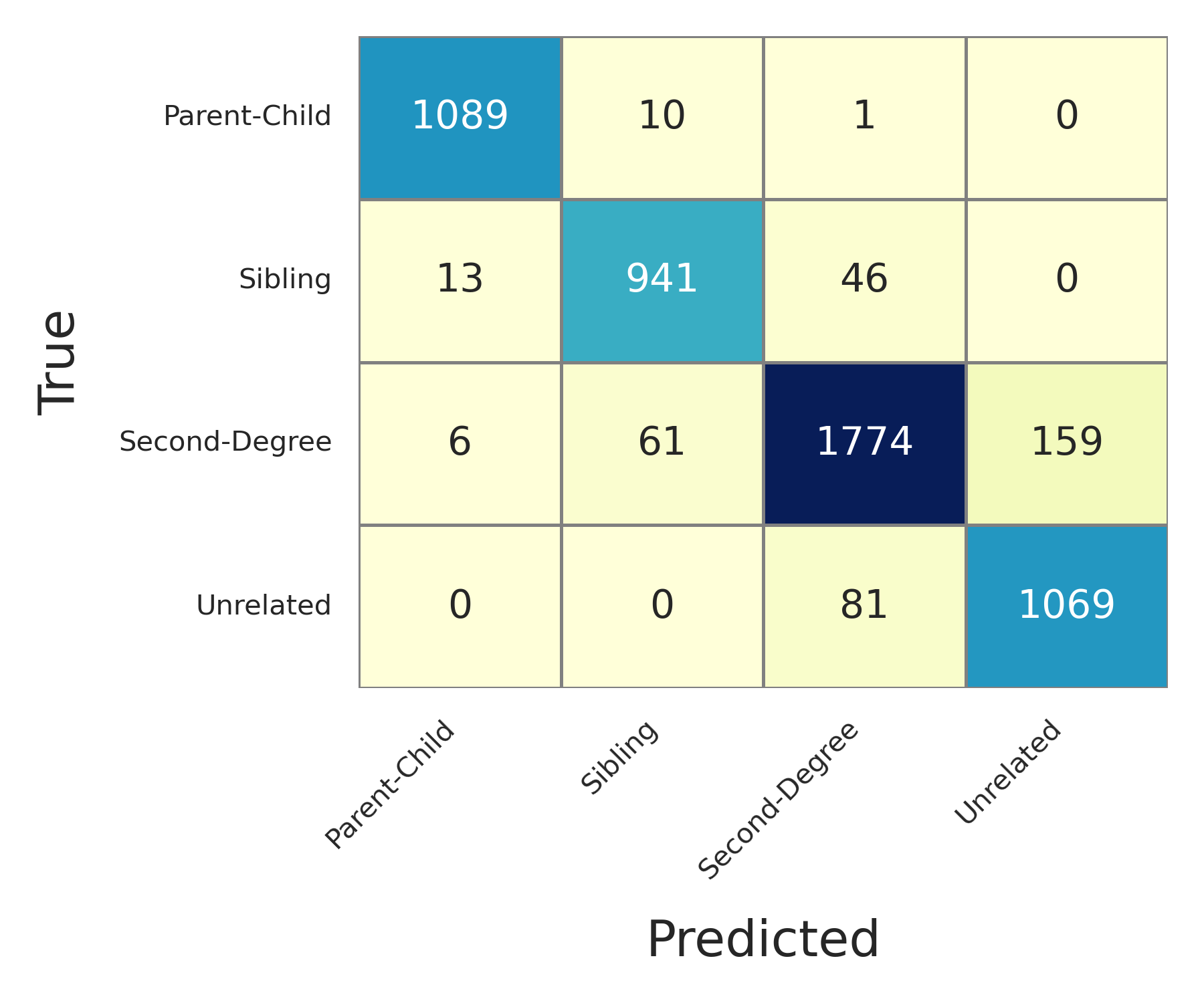
Notes: this table is the same as Table 5 in the main text.

CEU, MGD=30cM, MAF=0.4; simu error= 0.01; LR calculation error = 0.001

Accuracy = 0.9110; F1 Score = 0.9114


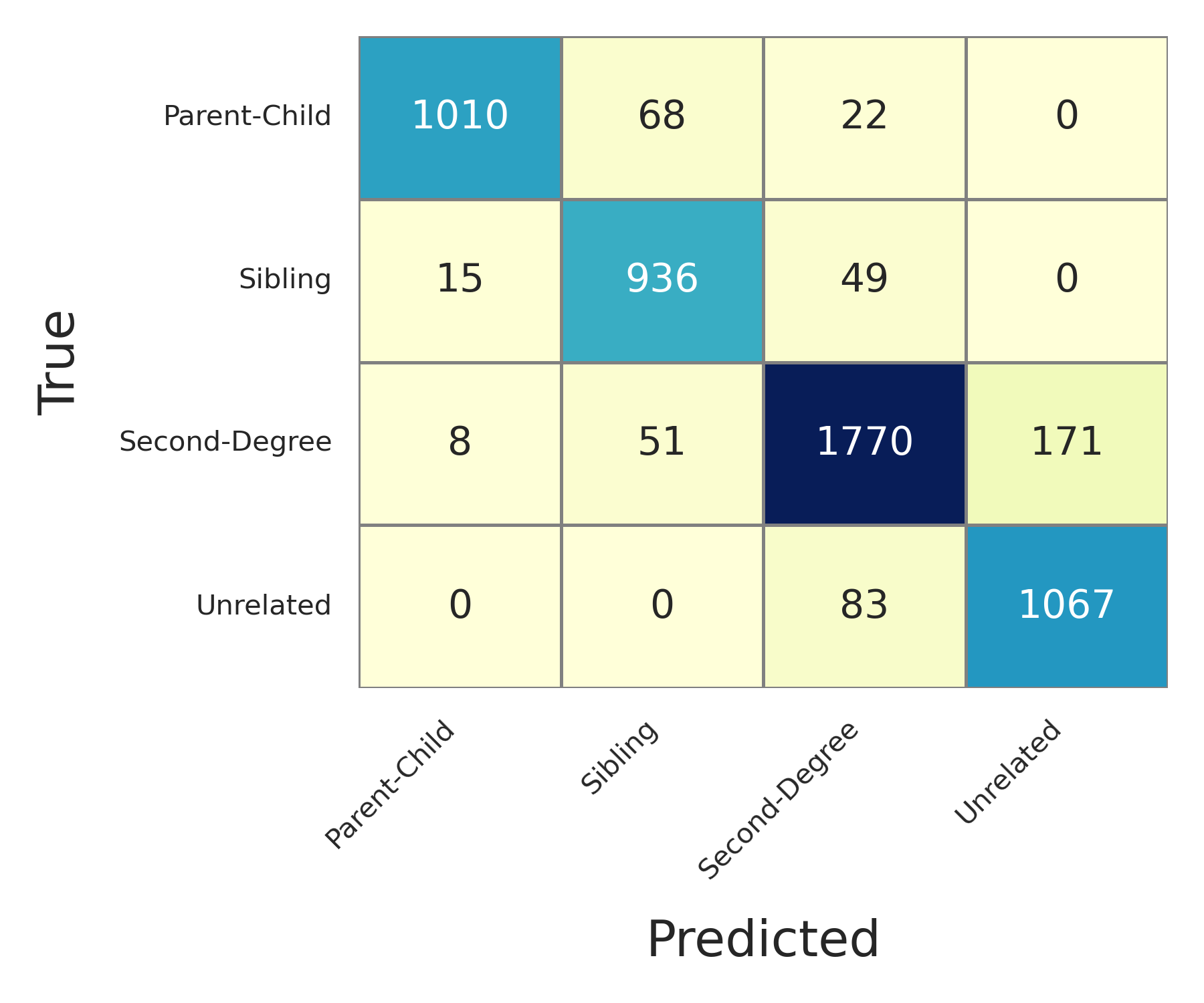


CEU, MGD=30cM, MAF=0.4; simu error= 0.01; LR calculation error = 0.01

Accuracy = 0.9198; F1 Score = 0.9198


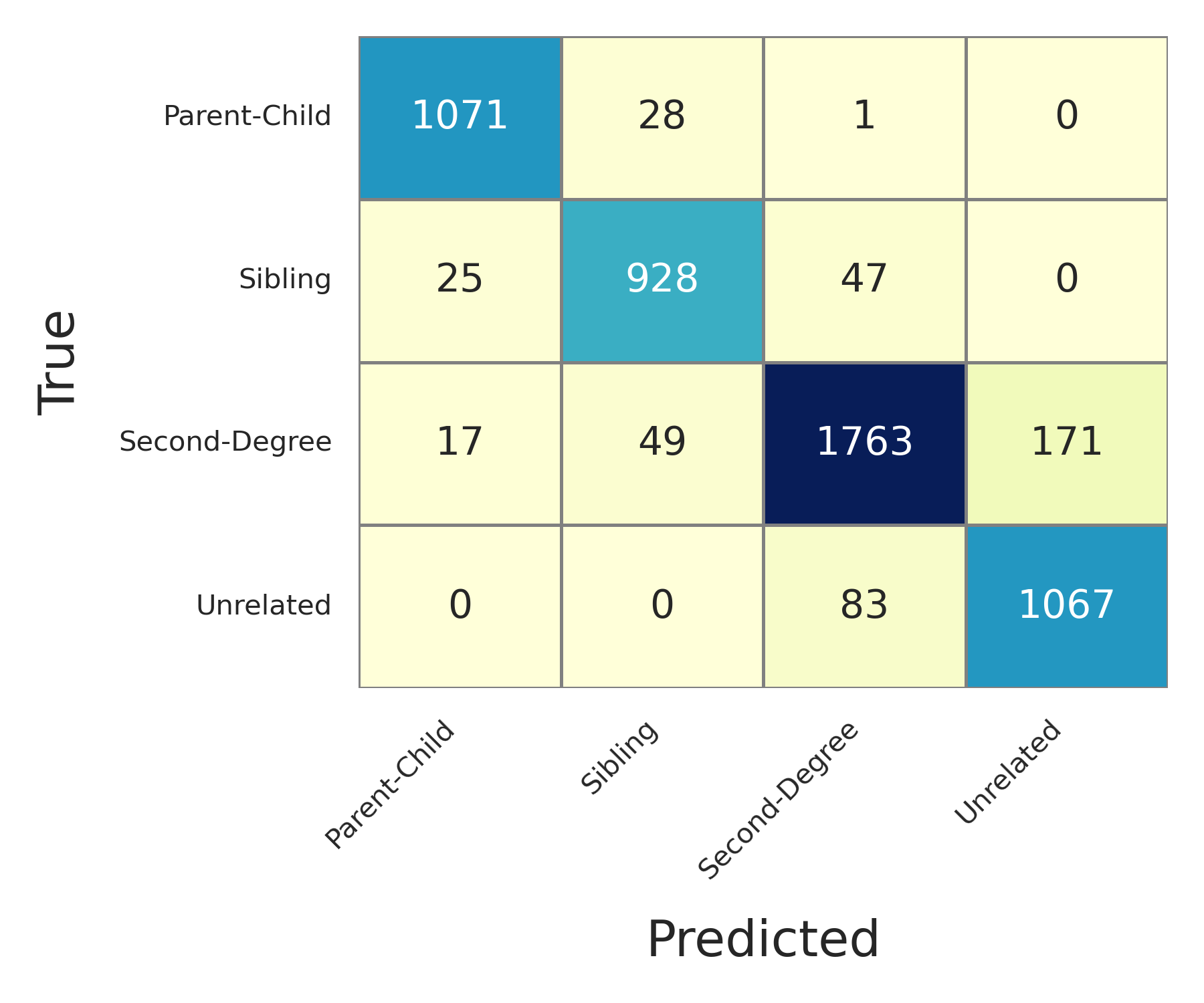


CEU, MGD=30cM, MAF=0.4; simu error= 0.05; LR calculation error = 0.001

Accuracy = 0.7907; F1 Score = 0.7805


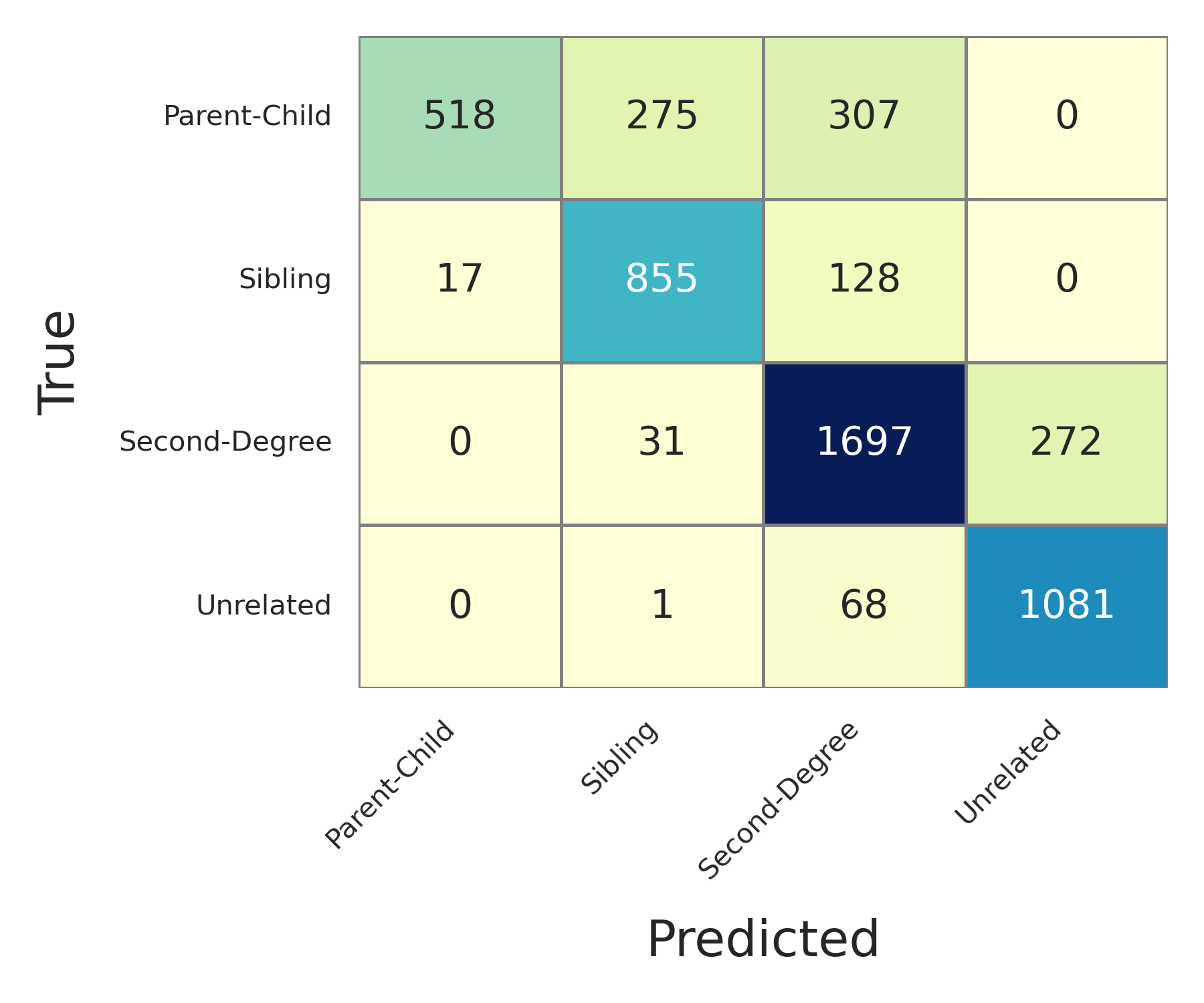


CEU, MGD=30cM, MAF=0.4; simu error= 0.05; LR calculation error = 0.05

Accuracy = 0.8663; F1 Score = 0.8660


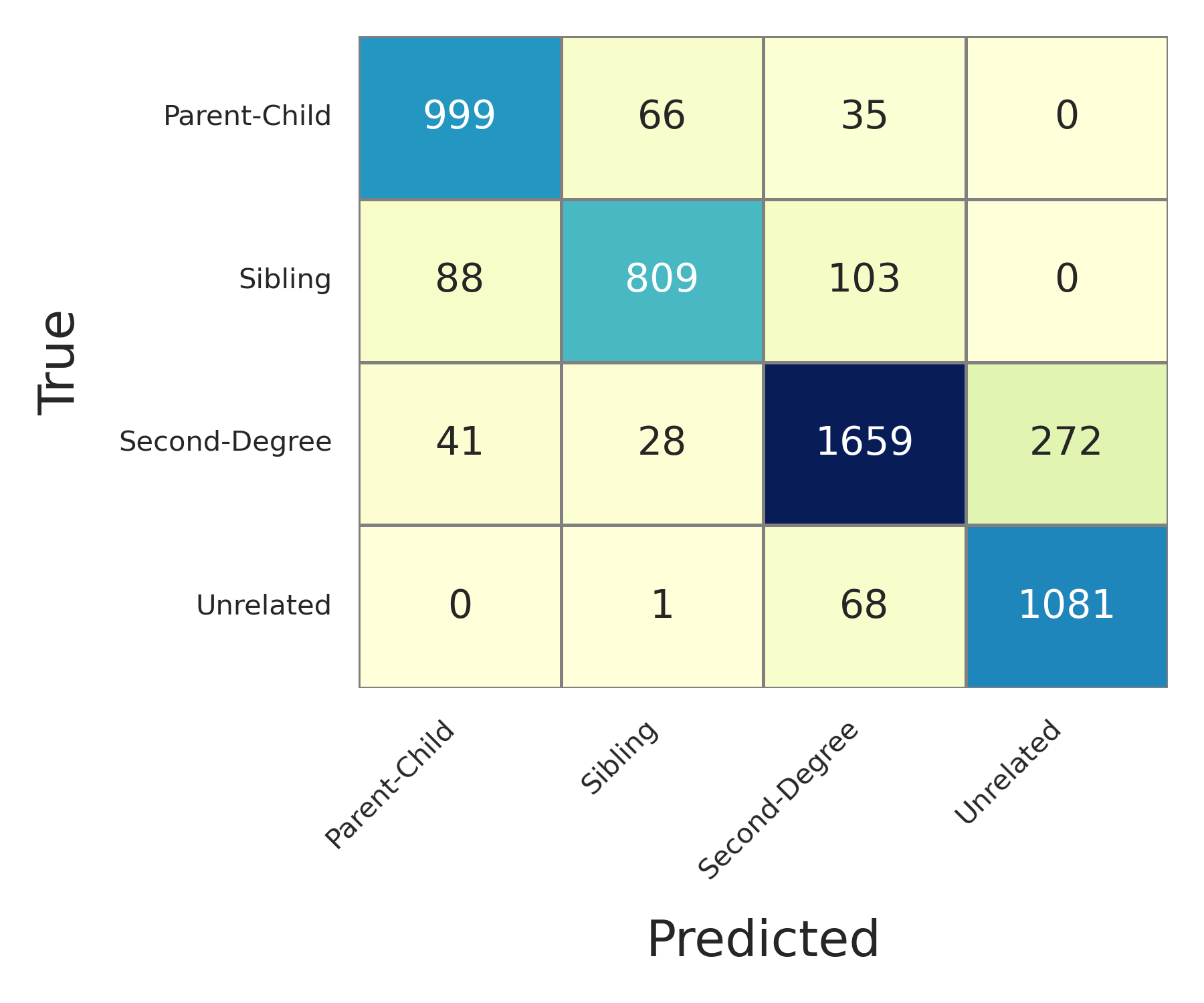

Supplement: Supplementary file 1 [file Supplementaryfile1.docx]
